# Methotrexate Enhances Apoptosis of Transmembrane TNF-Expressing Cells Treated With Anti-TNF Agents

**DOI:** 10.3389/fimmu.2020.02042

**Published:** 2020-08-14

**Authors:** Qiaolei Wang, Daisuke Oryoji, Hiroki Mitoma, Yasutaka Kimoto, Masamichi Koyanagi, Kana Yokoyama, Masahiro Ayano, Mitsuteru Akahoshi, Yojiro Arinobu, Hiroaki Niiro, Koichi Akashi, Takahiko Horiuchi

**Affiliations:** ^1^Department of Internal Medicine, Kyushu University Beppu Hospital, Beppu, Japan; ^2^Department of Medicine and Biosystemic Science, Graduate School of Medical Sciences, Kyushu University, Fukuoka, Japan

**Keywords:** anti-TNF agent, methotrexate, rheumatoid arthritis, transmembrane TNF, apoptosis, cytotoxicity

## Abstract

**Background:**

Concomitant use of methotrexate (MTX) improves the clinical efficacy of anti-TNF agents in the treatment of rheumatoid arthritis (RA). We aimed to clarify the cytotoxic effect of MTX on transmembrane TNF (tmTNF)-expressing cells treated with anti-TNF agents.

**Methods:**

Jurkat T cells stably expressing tmTNF were used for the following experiments. Cytotoxicity induced by an anti-TNF agent (infliximab, adalimumab, or certolizumab pegol) with concomitant MTX were compared with that by MTX alone or by an anti-TNF agent alone using flow cytometry. Apoptosis-induction mediated by reverse signal through tmTNF, complement-dependent cytotoxicity (CDC), antibody-dependent cell-mediated cytotoxicity (ADCC), and antibody-dependent cellular phagocytosis (ADCP) were evaluated. Folic acid and Y-27632, a Rho kinase inhibitor, were used as inhibitors to study intracellular signaling pathway in apoptosis induced by MTX and anti-TNF agents.

**Results:**

Apoptosis of tmTNF-expressing cells was significantly increased by the concomitant administration of MTX and an anti-TNF agent, compared with MTX alone or an anti-TNF agent alone. The apoptosis induction by concomitant MTX was most pronounced in infliximab-treatment. Reverse signal transduction, but not CDC or ADCC/ADCP, was responsible for the coordinate effect of MTX and an anti-TNF agent on tmTNF-expressing cells. Folic acid inhibited MTX-mediated apoptosis, while Y-27632 suppressed JNK activation and infliximab-induced apoptosis via revere signal through tmTNF.

**Conclusion:**

The apoptotic effect was enhanced by combination of MTX and an anti-TNF agent in tmTNF-expressing cells. The intracellular pathways induced by MTX and anti-TNF agents seem to be independent. These findings might explain at least in part improved the clinical response upon co-therapy of MTX and an anti-TNF agent in RA.

## Introduction

Tumor necrosis factor (TNF)-α is a pro-inflammatory cytokine that exerts pleiotropic effects on various type of cells and plays a major role in the pathogenesis of inflammatory diseases, such as rheumatoid arthritis (RA) (1–3). TNF-α -blocking therapies enable physicians to achieve remission or low disease activity in patients with RA.

Transmembrane TNF (tmTNF), a precursor from of soluble TNF, mediates its biological functions in a cell-to-cell contact manner. TmTNF binds to TNF receptor type 1 or 2 on cell surface of target cells as a ligand. In addition, tmTNF receives outside-to-inside (reverse) signals as a receptor back to tmTNF-expressing cells. Soluble TNF is a cleaved product of tmTNF via metalloproteinases on the cell surface and acts in distant places from TNF-producing sites (4). Anti-TNF agents bind to both of soluble TNF and tmTNF, resulting in neutralization of their biological activities (5). Moreover, anti-TNF agents induce cell death to tmTNF-expressing cells via the binding to tmTNF (6–8). Destruction of tmTNF-expressing cells via anti-TNF agents is mediated by antibody-dependent cellular cytotoxicity (ADCC), complement dependent cytotoxicity (CDC), and apoptosis via reverse signals through tmTNF (4, 7, 9).

In the treatment of RA, it is important to improve the disease activity and suppress the progression of joint damages. In the EULAR and ACR recommendations, methotrexate (MTX) is the anchor drug in the treatment of RA (10, 11). MTX shows anti-inflammatory effects (12) and suppresses the bone destruction (13), although its mechanism of actions has not been fully clarified (14). The biologics such as anti-TNF agents are used for the patients with inadequate responses to MTX. The concomitant use of MTX and an anti-TNF agent has been an important strategy in the treatment of RA. When an anti-TNF agent is administered, MTX is recommended to be used in combination, which can lead to a better clinical response than the monotherapy of MTX or an anti-TNF agent (15–18). Notably, the combination of MTX and several anti-TNF agents (infliximab and adalimumab) shows more desirable effects on suppressing joint damages compared with the monotherapy (19, 20).

The mechanisms that the concomitant use of MTX and an anti-TNF agent is superior to the monotherapy have not been clearly elucidated. There might be a number of possible mechanisms including abrogation of production of anti-drug antibodies (ADAb) and synergism on the disease process (21). Of these mechanisms, reduction of immunogenicity of anti-TNF agents by MTX seems to be well documented. Concomitant use of MTX is proved to attenuate the formation of ADAb during the treatment with anti-TNF agents in RA and in other chronic inflammatory diseases (21–23). Generation of ADAb has been known to be an important mechanism for loss of clinical responses over time in RA. Combination with MTX may improve the drug efficacy of IFX and ADA by reducing the generation of anti-IFX antibody or anti-ADA antibody in RA patients (24). It is possible, however, that other actions of mechanisms are involved in the combined effect of MTX and an anti-TNF agent.

In the present study, we aimed to show the combined effect of MTX and an anti-TNF agent on TNF-producing cells. By using tmTNF-expressing Jurkat cell line, we demonstrated that co-treatment of MTX and an anti-TNF agent enhanced apoptosis additively or synergistically compared to the monotherapy of these agents. MTX-mediated apoptosis was caused by inhibition of folic acid metabolism, while anti-TNF-mediated apoptosis was dependent on the Rho GTPase-JNK pathway via reverse signal through tmTNF. The intracellular signaling pathway mediated by MTX and an anti-TNF agent were independent.

These results suggest that simultaneous administration of MTX and an anti-TNF agent enhances apoptosis in tmTNF-expressing cells compared with the monotherapy of MTX or an anti-TNF agent. This may explain at least in part why MTX improves the clinical response mediated by an anti-TNF agent in RA.

## Materials and Methods

### Cell Culture

Transmembrane TNF-expressing Jurkat cells were established by a stable transfection of human pro-TNF-α resistant to TACE-mediated cleavages as described previously (25). Cells were maintained in RPMI-1640 supplemented with 10% heat-inactivated fetal bovine serum, 100 U/ml penicillin and 100 μg/ml streptomycin and were incubated at 37°C in a 5% CO_2_-humidified atmosphere.

### Antibodies and Reagents

Infliximab (IFX), Etanercept (ETN), Certolizumab Pegol (CZP) were provided by Mitsubishi Tanabe Pharma Corporation (Osaka, Japan), Takeda Pharmaceutical Company Limited (Osaka, Japan) and UCB Japan (Tokyo, Japan), respectively. Methotrexate and folic acid were obtained from Wako Chemicals (Osaka, Japan). Rho kinase inhibitor, Y27632, and PKH-26 were supplied from Sigma (St. Louis, MO, United States). TO-PRO-3 was from Thermo Fisher Scientific (Waltham, MA, United States). Rabbit anti-phospho JNK monoclonal antibody (mAb) and anti-JNK polyclonal antibody (Ab) were purchased from cell signaling technology (Danvers, MA, United States). Anti-GAPDH Ab was from Gene Tex (Irvine, CA, United States).

### Apoptosis Assay

Transmembrane TNF-transfected Jurkat cells were stimulated with 0.1 μM MTX for 20 h, 0.01 μM infliximab for 6 h or combination of 0.1 μM MTX and 0.01 μM Infliximab for 6 h after the 14 h-stimulation with 0.1 μM MTX. In case of ETN and CZP, cells were stimulated with 0.1 μM MTX for 24 h, 0.01 μM ETN/CZP for 12 h or combination of 0.1 μM MTX and 0.01 μM ETN/CZP for 12 h after 12 h-stimulation with 0.1 μM MTX. Apoptosis and cells death were detected by the flow cytometry using a MEBCYTO apoptosis Kit (MBL, Nagoya, Japan).

### Complement-Dependent Cytotoxicity (CDC)

Transmembrane TNF-expressing Jurkat cells were stimulated by 0.1 μM MTX for 25 h, 0.01 μM IFX for 1 h, or combination of 0.1 μM MTX and 0.01 μM IFX for 1 h after 24 h-stimulation with 0.1 μM MTX in the presence of 5% fresh or heat-inactivated human serum. Treated cells were stained with propidium iodide for 15 min in the dark and dead cells were evaluated by flow cytometry.

### Antibody-Dependent Cell-Mediated Cytotoxicity (ADCC)

Transmembrane TNF-expressing Jurkat cells were used as target cells and were labeled with membrane dye PKH26 (Sigma). Labeled target cells were suspended in culture medium at a concentration of 1 × 10^6^/ml. Peripheral blood mononuclear cells (PBMCs) were used as effector cells and were prepared from heparinized blood (obtained from healthy individuals) by centrifugation on Ficoll (GE Healthcare, Tokyo, Japan). After 100 μl of labeled target cells were incubated with or without 0.1 μM MTX in flat-bottomed 96-well-plates for 24 h, effector cells in 100 μl of culture medium were added at effector cells (E): target cells (T) ratio from 1:4 to 4:1 and incubated for 2 h with or without 0.01 μM IFX.

### Antibody-Dependent Cellular Phagocytosis (ADCP)

We used the Fcγ*RIIa*-H ADCP reporter assay (Promega, Madison, WI, United States) to detect ADCP activity. TmTNF-expressing Jurkat cells were used as the target cells to co-incubate with Fcγ*RIIa*-H-expressing effecter cells in a ratio of 1:1 and distributed on 96-well-plates (150 μ l with 5 × 10^4^ cells). The co-incubated cells were stimulated with 0.1 μ M MTX, 0.01 μ M IFX or combination of MTX and IFX for 6 h. The activation of ADCP is detected by the NFAT-RE-mediated luciferase activity of Fcγ*RIIa*-H-expressing effect cells results from target tmTNF-expressing cells bind to agents and the bioluminescent signal is quantified by using Luminoscan Ascent (Thermo Fisher Scientific).

### Apoptosis Assay in Human CD4^+^ T Cells

Human CD4^+^ T cells were isolated from healthy subjects using CD4^+^ T cells isolation kit (STEMCELL Technologies, Vancouver, Canada). purified CD4^+^ T cells were stimulated with pre-coated anti-CD3/28 Ab (Thermo Fisher Scientific) for 1 h. Activated cells were seeded on 96-well-plates with a density of 5 × 10^5^ cells/ml and cultured with RPMI 16040 supplemented with 10% FBS, 100 U/ml of penicillin, and 100 μl of streptomycin. Phytohemagglutinin (PHA, Sigma) was added at a final concentration of 5 μg/ml to co-cultured with 1 μM MTX, 1 μM an anti-TNF agent (IFX, ETN, or CZP), or MTX plus an anti-TNF agent for 48 h. Apoptotic cells were analyzed by Annexin V/PI staining.

### Western Blot

Transmembrane TNF-expressing Jurkat cells were stimulated with 0.01 μM IFX for 2 h in the presence (Rhoi-IFX) or absence (IFX) of 0.01 μM of Y-27632. Stimulated or unstimulated cells were lysed in SDS sample buffer. Separated proteins by electrophoresis were then transferred onto nitrocellulose membrane. Then the membrane was incubated with rabbit anti-phospho SAPK/JNK (1:1000) or anti-JNK (1:1000) in Tris-Buffered Saline-Tween (TBS-T) with 5% bovine serum albumin at 4°C overnight. The membrane was washed three times with TBS-T and incubated with HRP-conjugated anti-rabbit IgG at room temperature for 1 h. The membrane was visualized with ECL western blotting detection regents (GE Healthcare Japan).

### Statistical Analysis

Values of all results were performed as mean ± SEM. Significantly evaluation was conducted by Mann-Whitney *U* test/student *t*-test using Graph pad software.

## Results

### Co-administration of MTX and an Anti-TNF Agent Induced Apoptosis Additively or Synergistically in TmTNF-Expressing Jurkat Cells

**FIGURE 1:**
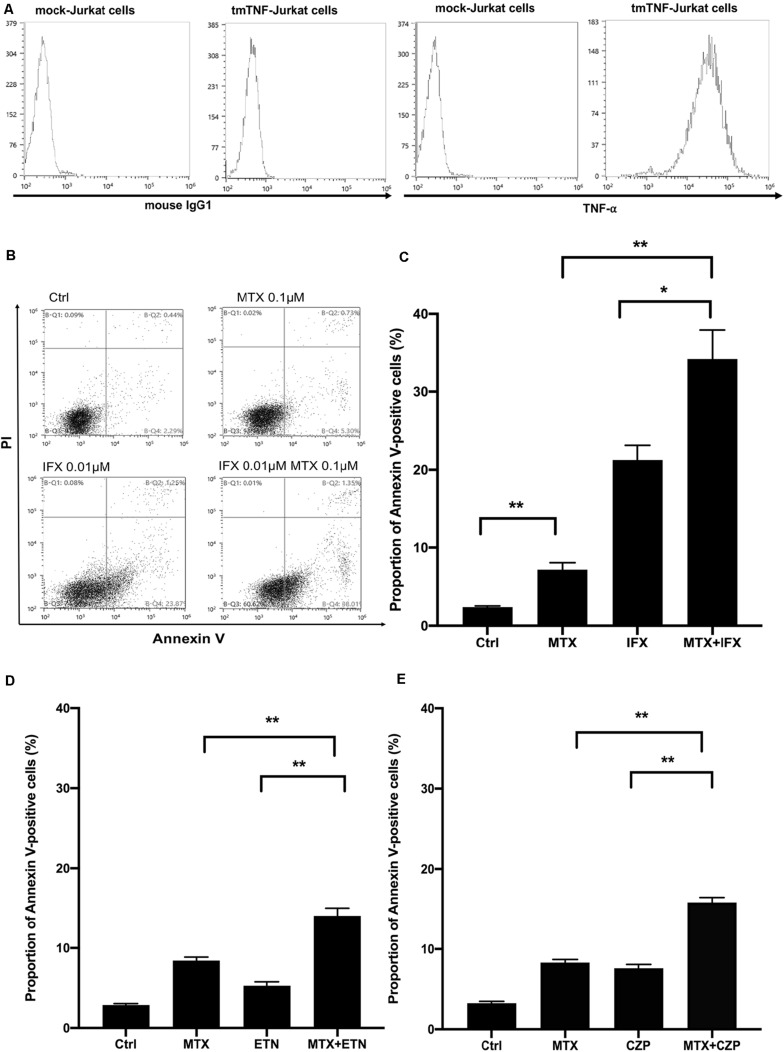
Concomitant treatment of MTX with an anti-TNF agent enhanced apoptosis of tmTNF-expressing cells. **(A)** Mock or tmTNF-transfected Jurkat cells were stained with FITC-conjugated mouse anti-TNF-α mAb or control mouse IgG and analyzed by flow cytometry. The histograms show the cell surface expression levels of tmTNF. **(B)** TmTNF-Jurkat cells were incubated with 0.1 μM of MTX for 20 h, 0.01 μM of infliximab (IFX) for 6 h, or combination of MTX and IFX for 6 h after MTX for 14 h. Stimulated cells were stained with FITC-conjugated Annexin V and propidium iodide (PI), and the apoptotic cells were detected by flow cytometry. **(C)** The proportion of Annexin V-positive apoptotic cells was indicated. Values are mean (±SEM) of each group (*n* = 5/group). **(D)** TmTNF-expressing Jurkat cells were incubated with 0.1 μM MTX for 24 h, 0.01 μM ETN for 12 h, or combination of MTX and ETN for 12 h after MTX for 12 h. The proportion of Annexin V-positive cells was indicated. Values are mean (±SEM) of each group (*n* = 6/group). **(E)** TmTNF-expressing Jurkat cells were incubated with 0.1 μM MTX for 24 h, 0.01 μM CZP for 12 h, or combination of MTX and CZP for 12 h after MTX for 12 h. The proportion of Annexin V-positive cells was indicated. Values are mean (±SEM) of each group (*n* = 6/group). *p < 0.05, **p < 0.01, Mann-Whitney *U* tests.

In order to clarify the mechanisms of improved clinical efficacy induced by concomitant treatment of MTX and an anti-TNF agent other than inhibiting generation of ADAb, we took note of the apoptotic effect mediated by reverse signal through tmTNF in tmTNF-expressing cells. To examine the effects of concomitant administration of MTX and an anti-TNF agent on apoptosis, we used Jurkat cells constitutively expressing tmTNF on their surface (5, 6, 9). Expression levels of tmTNF on these cells were confirmed by flow cytometry (Figure 1A). These cells were cultured with MTX and/or an anti-TNF agent, thereafter proportion of Annexin V-positive apoptotic cells was evaluated by flow cytometry.

At first, we studied for apoptosis induced by MTX alone, IFX alone and MTX plus IFX in tmTNF-expressing cells to see their combined cytotoxic effects. IFX is a chimeric anti-TNF full IgG1. Treatment with MTX alone for 20 h induced apoptosis in 7.2% of tmTNF-expressing cells, on the other hand untreated control showed apoptosis in 2.4% of these cells (*p* < 0.01). Stimulation with IFX alone for 6 h also significantly increased apoptotic cells to 21.3% compared to control (*p* < 0.01). Of note, apoptotic cells dramatically increased up to 34.2% under co-administration of MTX and IFX (20 h of MTX, and IFX was present for the last 6 h) (Figures 1B,C). Therefore, synergistic apoptotic effect was observed in co-administration of MTX and IFX.

Subsequently, to investigate whether this apoptosis-inducing effect differs among anti-TNF agents, similar experiments were carried out using other anti-TNF agents; etanercept (ETN), a fusion protein of extracellular domain of TNF receptor 2 and IgG1-Fc, and certolizumab pegol (CZP), a PEGylated Fab’ fragment of the anti-TNF antibody. As these two anti-TNF agents are much weaker in inducing apoptosis of tmTNF-expressing cells than IFX, the incubation time in the presence of these agents was prolonged from 6 to 12 h. After 24 h of incubation with MTX alone, Annexin V-positive apoptotic cells were 8.4% of the tmTNF-expressing cells and apoptotic cells in control were 2.8%. Treatment of ETN alone for 12 h induced apoptosis in 5.3% of the tmTNF-expressing cells. In the situation of co-stimulation, the percentage of apoptotic cells induced by co-stimulation of MTX and ETN was 14.0%, approximately equal to the sum of percentages of apoptotic cells by MTX alone and ETN alone (Figure 1D). Furthermore, similar additive effect with MTX was observed in CZP treatment. Proportions of apoptotic cells in control, MTX alone, CZP alone, and MTX plus CZP were 3.3, 8.3, 7.6, and 15.8%, respectively (Figure 1E). Taken together, MTX showed an additive apoptotic effect when co-stimulated with ETN or CZP in tmTNF-expressing T cells.

### The Additive Effect via Combination of MTX and an Anti-TNF Agent Were Observed in Neither Complement-Dependent Cytotoxicity, Antibody-Dependent Cell-Mediated Cytotoxicity, nor Antibody-Dependent Cellular Phagocytosis

To examine whether synergistic or additive effects between MTX and an anti-TNF agent are observed in cytotoxic assays other than apoptosis by reverse signal through tmTNF, we performed CDC and ADCC/ADCP assay.

**FIGURE 2:**
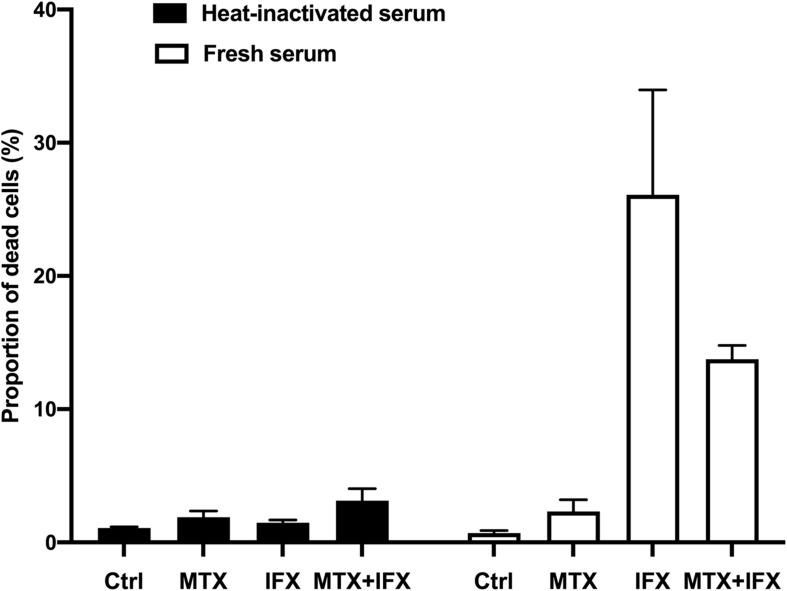
Complement-dependent cytotoxicity induced by IFX in co-stimulation with MTX. TmTNF-expressing Jurkat cells were stimulated with 0.1 μM MTX for 25 h, 0.01 μM IFX for 1 h, or combination of MTX and IFX for 1 h after MTX for 1 h, with 5% human fresh serum (white bar) or heat-inactivated serum (black bar). Treated cells were stained with PI and dead cells were detected by flow cytometry. The proportion of dead cells was indicated. Values are mean (±SEM) of each group (*n* = 3/group).

Transmembrane TNF-expressing cells were cultured with MTX for 25 h, IFX for 1 h or MTX plus IFX for 1 h after undergone 24 h of MTX treatment in the medium containing fresh or heat-inactivated serum to evaluate CDC activities (Figure 2). IFX-induced prominent cell death in human fresh serum, but not in heat-inactivated serum, indicating that IFX has CDC activity against tmTNF-expressing cells as previously reported (9). Co-administration of MTX and IFX did not exert any additional effects to cell death induced by IFX alone, hence the additive cytotoxic effect of co-administration of MTX and IFX was not observed in CDC assay. Similarly, a combined effect of MTX/ETN or MTX/CZP was not shown in CDC assay (data not shown).

**FIGURE 3:**
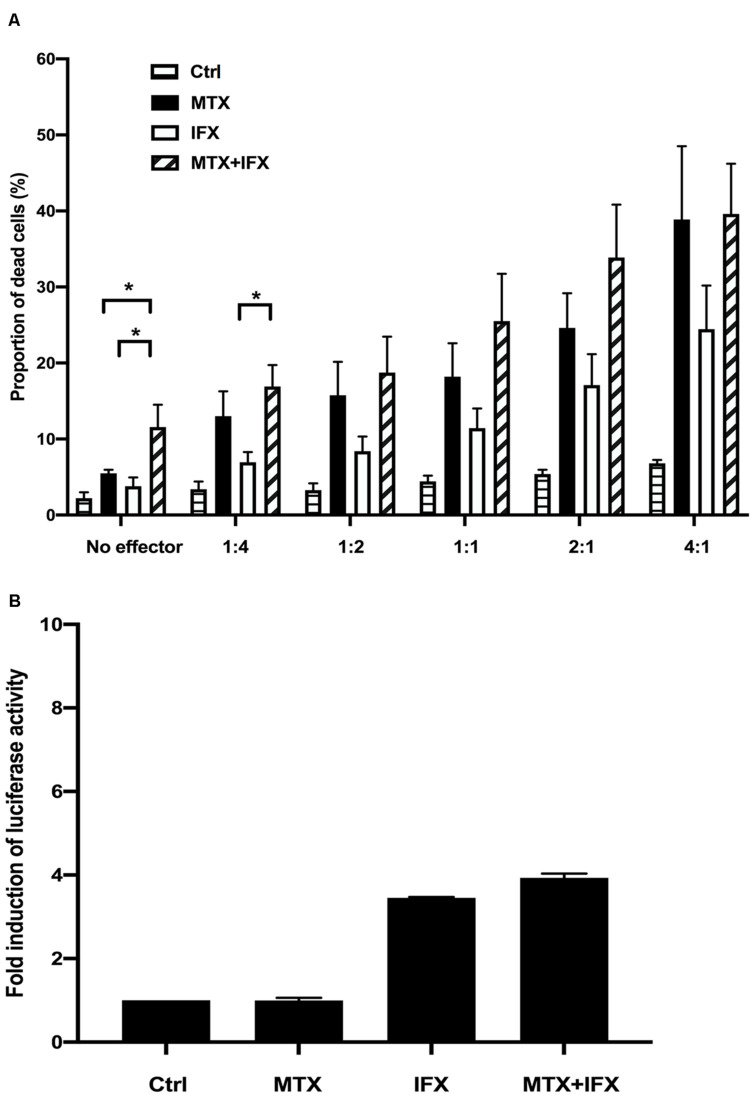
Antibody-dependent cell-mediated cytotoxicity and antibody-dependent cellular phagocytosis induced by IFX in co-stimulation with MTX. **(A)** TmTNF-expressing Jurkat cells were used as target cells and labeled with membrane dye PKH26. Peripheral blood mononuclear cells (PBMCs) were used as effector cells and co-incubated with target cells on flat-bottom plates. The ratios of effector cells and target cells was from 1:4 to 4:1. Cells were incubated with MTX for 24 h, IFX for 2 h, or combination of MTX and IFX for 2 h after MTX for 24 h. As a control, target cells were incubated with effector cells without MTX and IFX. Treated cells were stained with TO-PRO-3 iodide. TO-PRO-3 iodide-positive dead cells in PKH26-positive target cells were detected by flow cytometry. Proportion of dead cells in target cells was indicated. Values are mean ± SEM; *n* = 4/group. **(B)** TmTNF Jurkat cells (2 × 10^4^ in 150 μl/well) were co-incubated with FcγRIIa-H effector cells (2 × 10^4^ in 150 μl/well) and stimulated with 0.1 μM MTX, 0.01 μM IFX, or MTX+IFX for 6 h. Treated cells were cultured with luciferase assay buffer for 20 min and the luminous cells were detected by a luminometer. Values are mean ± SEM; *n* = 3/group. *p < 0.05, Mann-Whitney *U* tests.

We next investigated ADCC activities when tmTNF-expressing cells were cultured with MTX and/or IFX. TmTNF-expressing cells were used as target cells pre-incubated with or without MTX. They were co-cultured with PBMCs (effector cells) in the ratios from 1:4 to 4:1 with or without IFX for 2 h. As the E: T ratio was higher, ratio of cell death in target cells gradually increased. At the E:T ratio of 4:1, we found nearly 16% more dead cells in target cells treated with IFX and effector cells compared to effector cells alone (Figure 3A). The cell death in target cells treated with MTX also increased even in the absence of IFX as the ratio of co-cultured effector cells became higher (Figure 3A). The cytotoxic effect of MTX seemed to be augmented in the presence of PBMCs. Next, we examined the effect of MTX and IFX in ADCC circumstance. Although ADCC by IFX in the presence of MTX is gradually elevated according to the increase of the E: T ratio, the combined effect of MTX and IFX was not observed when compared with the cytotoxic effects of MTX alone or IFX alone (Figure 3A). Thus, we conjectured that synergic or additive effect of MTX and IFX was not observed in ADCC assay in tmTNF-expressing Jurkat cells.

The conception of ADCP (antibody-dependent cellular phagocytosis) has been proposed recently whose mechanism is similar with ADCC. In response to antibody therapies, ADCP is mediated by macrophages via the recognition of Fc portion of antibodies. We used FcγRIIa-H effector cells which contain the Histidine at amino acid 131 for ADCP reporter assay to co-culture with tmTNF-expressing Jurkat cells. The co-incubated cells were stimulated with MTX alone, IFX alone or MTX plus IFX for 6 h. The FcγRIIa-H effector cells bound to Fc domain of IFX resulted in the signaling of NFAT-RE-mediated luciferase activity. IFX-treatment induced about 3.5 times activation of ADCP compared with the control group. Definitely, MTX alone did not show any abilities to mediate phagocytosis. The additive effect of MTX and IFX was not observed in the ADCP activity (Figure 3B).

Taken together, these results suggested that the additive cytotoxic effect induced by co-administration of MTX and IFX was displayed in neither CDC, ADCC, nor ADCP.

### Folic Acid Inhibited MTX-Induced Apoptosis

**FIGURE 4:**
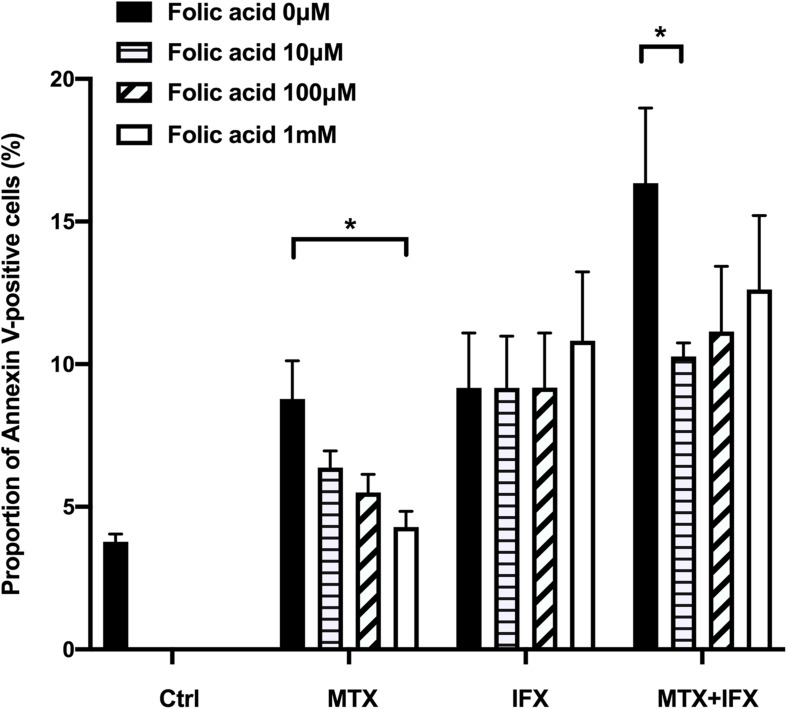
MTX-induced apoptosis was mediated by folate cycle inhibition. TmTNF-expressing Jurkat cells were incubated with 0.1 μM MTX for 24 h, 0.01 μM IFX for 4 h or combination of MTX and IFX for 4 h after MTX for 20 h, in the presence of 0, 10, 100, and 1000 μM folic acid. Proportion of Annexin V-positive cells were indicated. Values are mean (±SEM) of each group (*n* = 4/group). *p < 0.05, Mann Whitney *U* tests.

Methotrexate inhibits dihydrofolate reductase, resulting in folate cycle inhibition. Folic acid is known to protect cells from anti-folate effects induced by MTX. Supplementation of folic acid into the culture medium dose-dependently reverted cell death induced by MTX alone from 8.8% to 4.3% (Figure 4). On the other hand, folic acid did not inhibit apoptosis induced by IFX. Folic acid partially suppressed cell death induced by co-stimulation of MTX and IFX (Figure 4). Therefore, anti-folate effect is required for MTX-induced apoptosis, but not for apoptosis mediated by reverse signal through tmTNF via IFX.

### A Rho Kinase Inhibitor Prevents Apoptosis and JNK Activation Induced by IFX in TmTNF-Expressing Cells

We hypothesized that the synergistic effect on induction of apoptosis via combination of MTX and IFX is elicited by two distinct and independent pathways; cytotoxic effect of MTX and reverse signal through tmTNF. To investigate this hypothesis, we aimed to elucidate the intracellular pathway of reverse signal through tmTNF in more details. It has been reported that the induction of apoptosis via reverse signal requires the activation of JNK (6), but the upstream mediators of the JNK pathway have not been clarified.

**FIGURE 5:**
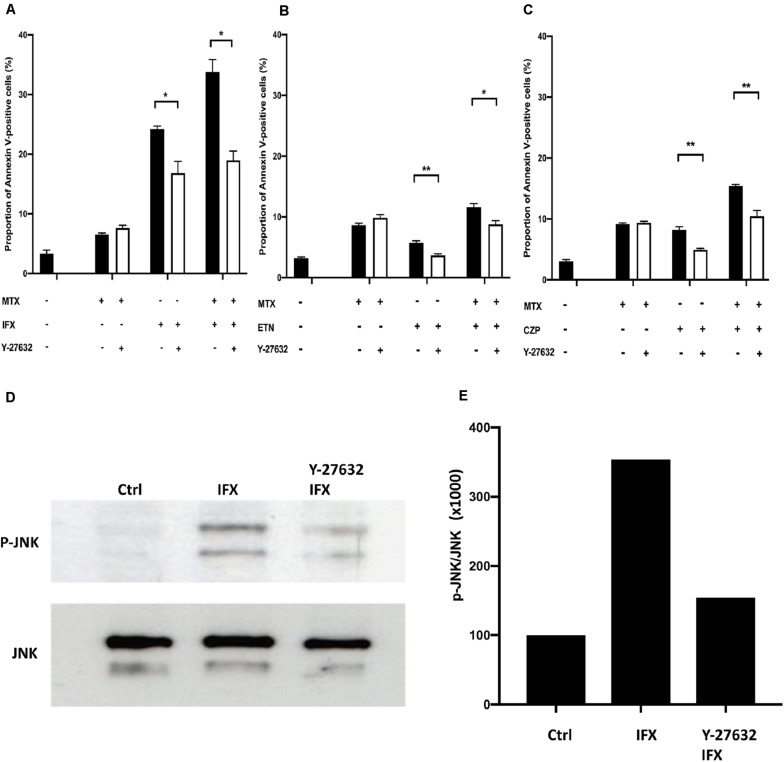
Infliximab-induced apoptosis was mediated by activation of Rho kinase. **(A)** TmTNF-expressing Jurkat cells were incubated with 0.1 μM MTX for 20 h, 0.01 μM IFX for 6 h, or combination of MTX and IFX for 6 h after MTX for 14 h, in the presence (white bar) or absence (black bar) of 0.01 μM of Rho kinase inhibitor (Y-27632). Apoptotic cells were analyzed by flow cytometry using Annexin V staining. The proportion of Annexin V-positive cells was indicated. **(B)** TmTNF- expressing Jurkat cells were stimulated with 0.1 μM MTX for 24 h, 0.01 μM ETN for 12 h, or combination of MTX and ETN for 12 h after MTX for 12 h with or without Y-27632. **(C)** TmTNF-expressing Jurkat cells were stimulated with 0.1 μM MTX for 24 h, 0.01 μM CZP for 12 h, or combination of MTX and CZP for 12 h after MTX for 12 h with or without Y-27632. Values are mean ± SEM; *n* = 4/group in **(A)** and *n* = 5/group in **(B,C)**. *p < 0.05, **p < 0.01, Mann Whitney *U* tests. **(D,E)** TmTNF-expressing Jurkat cells were stimulated with 0.01 μM IFX for 2 h in the presence (Rho-i + IFX) or absence (IFX) of 0.01 μM of Y-27632. Stimulated cells or unstimulated cells (Ctrl) were lysed in lysis buffer and the levels of phosphorylated JNK (p-JNK) was analyzed in western blotting **(D)**. The ratio of p-JNK: total JNK is indicated in **(E)**.

We focused on Rho GTPase, including Rho, CDC42, and RAC, which are reported to be mediators upstream of JNK in T lymphocytes (26), and performed experiments using the inhibitor of Rho GTPase. Y-27632, a Rho kinase inhibitor, was used to treat cells stimulated by MTX, IFX or MTX plus IFX. The proportion of IFX-induced apoptotic cells was significantly decreased by Y-27632 from 24.2 to 16.8% (*p* < 0.05), whereas there were no effects on MTX-induced apoptosis (Figure 5A). In the case of co-stimulation of MTX and IFX, inhibition of Rho significantly decreased the percentage of apoptotic cells from 33.8 to 19.0% (*p* < 0.05). Y-27632 also inhibited apoptosis induced by ETN from 5.7 to 3.7% (Figure 5B) and by CZP from 8.2 to 4.9% (Figure 5C). These results suggest that Rho is required for induction of apoptosis via reverse signal through tmTNF.

We next examined whether JNK was actually inhibited by Y-27632. As shown in Figures 5D,E, phosphorylated JNK was observed in IFX-treated tmTNF cells. The Rho inhibitor, Y-27632, significantly decreased the phosphorylation of JNK. These results suggest that IFX activates Rho GTPase via reverse signal through tmTNF and then activated Rho GTPase induces phosphorylation of JNK, which leads to the induction of apoptosis.

### Additive Effect by Cooperation of MTX and an Anti-TNF Agent in Human CD4^+^ T Cells

**FIGURE 6:**
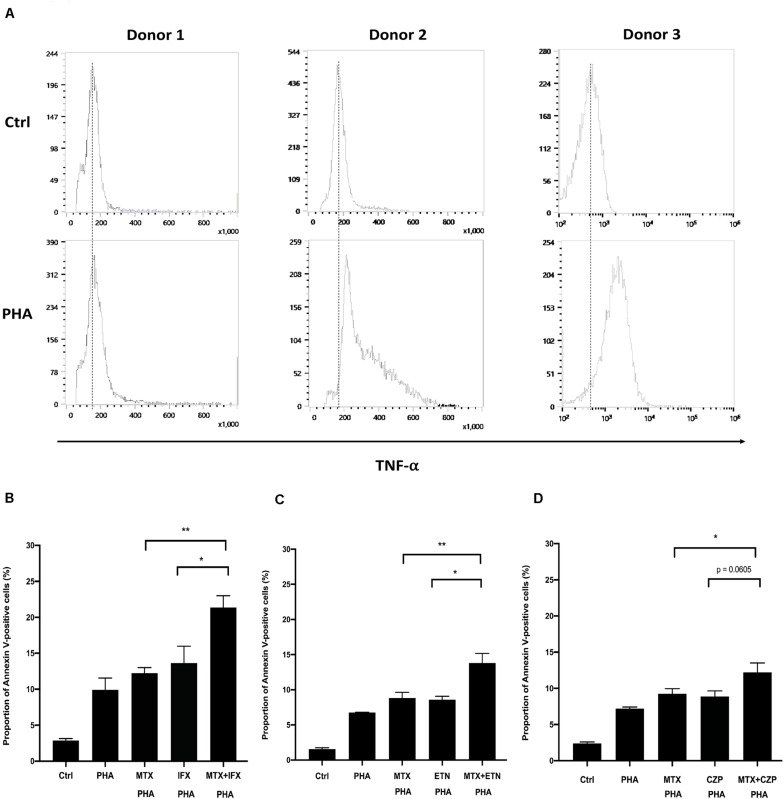
Concomitant treatment of MTX with an anti-TNF agent enhanced apoptosis of activated human CD4^+^ T cells. **(A)** CD4^+^ T cells were cultivated in the 96-well plate and activated by anti-CD3/28 Ab for 1 h. And then, cells were stimulated with 5 μg/ml PHA for 48 h. Cells were stained with FITC-conjugated anti-TNF and expression levels of TNF on the cells surface was detected by flow cytometry. **(B)** CD4^+^ T cells were untreated (Ctrl) or treated with PHA and stimulated with 1 μM MTX, 1 μM IFX, or combination of MTX and IFX for 48 h. The stimulated cells were stained with FITC-conjugated Annexin V and PI, and the apoptotic cells were detected by flow cytometry. **(C,D)** Similar experiments were performed with ETN **(C)** or CZP **(D)** instead of IFX. Values are mean ± SEM; *n* = 5/group, *p < 0.05, **p < 0.01, Paired *t*-test.

We finally examined whether the combined cytotoxic effect of MTX and an anti-TNF agent in reverse signal through tmTNF is also observed in primary human T lymphocytes. CD4^+^ T cells were purified from healthy donors and stimulated with anti-CD3/CD28 antibody for 1 h. Treatment with 5 μg/ml of phytohemagglutinin (PHA) for 48 h induced TNF-α expression on the surface of CD4^+^ T cells (Figure 6A). Apoptosis was observed in approximately 10% of cells after 48 h of PHA-stimulation. MTX, IFX, or MTX plus IFX were added in this assay. MTX or an anti-TNF agent showed tendency of increasing apoptosis of PHA-treated cells, but the difference was not statistically significant compared to untreated control. On the other hand, combination of MTX and IFX significantly increased apoptosis and showed an additive effect (Figure 6B). ETN and CZP were also examined and showed similar tendency with IFX (Figures 6C,D). However, the cytotoxic effect of IFX under concomitant use of MTX was more prominent compared with that of ETN or CZP. Therefore, the combined apoptotic effect of MTX and an anti-TNF agent via reverse signal through tmTNF was also observed in primary CD4^+^ T cells.

## Discussion

In the present study, we showed combined effects of MTX and anti-TNF agents, IFX, ETN, or CZP on the apoptosis of tmTNF-expressing cells. The combined effects were most prominent and synergistic in case of IFX. In the tmTNF-expressing cells, MTX and anti-TNF agents are likely to exert their apoptotic effects by two distinct pathways. Apoptosis by MTX was suggested to be dependent on folate cycle inhibition, while that by IFX was on Rho GTPase activation followed by JNK activation.

Etanercept or CZP-induced apoptosis was much weaker than IFX. Three monoclonal anti-TNF full IgG1 antibodies, IFX, adalimumab, and golimumab, almost equally induces apoptosis in tmTNF-expressing cells (8). Considering full antibodies have two Fab domains, they are able to bind two trimers of tmTNF on the cell surface and cross-link tmTNF. On the other hand, ETN or CZP bind only one trimer of tmTNF, thus they are not able to cross-link tmTNF. By cross-linking ETN with an anti-human IgG antibody, ETN-induced apoptosis is actually enhanced in tmTNF-expressing cells (6).

The coordinate apoptotic effect of MTX and an anti-TNF agent on tmTNF-expressing cells might explain at least in part the essential role of MTX to perform at the full potential of anti-TNF agents in clinical settings. MTX is considered to be effective for RA by various possible mechanisms (14). In this study, apoptosis-inducing effect of MTX was shown in tmTNF-expressing cells, which was established from Jurkat T cell line originated from the human leukemic T cell lymphoblast. In addition, a reduction in proliferation and an increase in apoptosis by MTX has been reported in mitogenically activated, but not resting, T lymphocytes from healthy subjects (27) and in activated T lymphocytes from RA patients (28). These findings indicate that induction of apoptosis in activated immune cells may be one of the important mechanisms of MTX for the treatment of RA.

Phytohemagglutinin, a kind of lectins found in plants, bind to cell membrane glycoproteins strongly to induce activation of T cells. It is known that PHA-activated T cells can express increased amount of tmTNF on the surface (25, 29). In the present study, we detected TNF-α on the surface of PHA-activated CD4^+^ T cells. As shown in tmTNF-expressing Jurkat cells, additive effect of MTX and anti-TNF agent was similarly observed in PHA-activated human CD4^+^ T cells. The magnitude of the apoptotic effect of MTX and anti-TNF agent in PHA-activated CD4^+^ T lymphocytes was weaker than that in tmTNF-expressing Jurkat cells. Apoptosis via MTX required progression of activated T cells to the S phase of the cell cycle (28). Jurkat T cells are highly proliferating and proportion of cells in S phase are approximately 30–50% (6), indicating that Jurkat cells are substantially sensitive to MTX-induced apoptosis. Although activated CD4^+^ T cells treated with MTX and anti-TNF agent showed lesser extent of apoptosis compared to Jurkat cells, the combined apoptotic effect of these two agents were significant and may contribute to suppress synovial inflammation with infiltration of activated CD4^+^ T cells.

In clinical settings of RA treatment, MTX and anti-TNF agent are basically used in combination. IFX has previously been reported for its apoptosis-inducing effect (6), and treatment with IFX resulted in an increased number of apoptotic cells in synovial tissue (30). It has been reported that the combination of MTX and IFX shows a significant effect of inhibiting bone destruction compared with MTX monotherapy in irrespective of the groups carrying different clinical disease activities (19). Our results suggest that simultaneous administration of MTX and IFX enhances apoptosis of activated immune cells expressing tmTNF in synovial tissues in RA patients compared with MTX or IFX monotherapy. This may explain some of the mechanisms of clinically reported effects on inhibition of joint damage.

The main effect of ETN is the neutralization of soluble TNF, but it also binds to tmTNF and modifies the functions of tmTNF. However, in this study, single administration of ETN did not show any significant apoptosis via reverse signal in tmTNF-producing cells. CZP also binds to soluble TNF and tmTNF. CZP plus MTX has better clinical response than MTX monotherapy in RA (16). The apoptosis-inducing effect has been confirmed in the present study, but the mechanism by which CZP induces apoptosis is unknown (8).

The mechanisms of coordinate effect between MTX and anti-TNF agent have been implicated by a number of mechanisms. Suppression of ADAb against anti-TNF agents is considered to be a possible mechanism. In fact, a recent review on immunogenicity across inflammatory diseases reported that the incidence of ADAb for IFX, ADA, GLM, and CZP are 0–83%, 0–54%, 0–19% and 21–52%, respectively (31). However, in the case of ETN, a fusion protein of TNFR2 and Fc portion of IgG, the incidence of ADAs is quite low, 0–13%. Moreover, ADAb for ETN were not neutralizing (31). Considering the combined effect of MTX plus ETN is observed both in clinical response and joint damage protection, suppression of ADAb by MTX might not be the sole effect for improvement of output of anti-TNF agent.

The influence of MTX on cytokine network in the presence of anti-TNF is unclear. A report describing MTX significantly reduced plasma IL-6, but not TNF (32). In contrast, after ex vivo activation, T cells from MTX-treated patients with RA have a reduced production of a number of cytokines including TNF compared with T lymphocytes from MTX-naïve patients (33). Our present data that concomitant administration of MTX and anti-TNF agent suppress tmTNF-expressing cells compared to a single agent might indicate that MTX not only suppress ADAb formation but also act coordinately with anti-TNF agents on the disease process.

The Rho proteins are the branches of Rho GTPase that are activated by the cycle switches between inactive GDP-bound and active GTP-bound conformation. The exchange of GDP to GTP is controlled by guanine nucleotide exchange factors (GEFs). There are 23 members in the Rho family in which three special members, RhoA, Cdc42 and Rac, have been researched for the functional characteristics in detail (34). The main roles of activated Rho GTPase in the cellular function includes action on actin and myosin filaments, cytoskeletal regulation and transduction of linking receptors to the activated protein. In addition, Rho GTPase can regulate some other signal pathways of cell polarity, cell cycle progression, apoptosis and response to stress (34). After binding of IFX to tmTNF, JNK is activated and eventually upregulation of Bax and Bak and activation of caspase 3, as well as cell cycle arrest occurs (6). Some members of Rho subfamily like Rac2, CDC42 have been reported to be responsible for JNK and p38 activation on cell growth and proliferation in T lymphocytes (26). In the present study, we found that the JNK activation is regulated by its upstream Rho GTPase in reverse signal through tmTNF.

We demonstrated that concomitant administration of MTX and anti-TNF agent to tmTNF-expressing cells enhances apoptosis additively or synergistically. The apoptotic effect by MTX was mediated by the inhibition folate cycle, while that by IFX was dependent on the Rho-JNK pathway via reverse signal through tmTNF. These results suggest that simultaneous administration of MTX and IFX enhances apoptosis induction of immune cells expressing tmTNF in rheumatoid arthritis compared with monotherapy. This may explain at least in part the coordinate clinical response by MTX and anti-TNF agent.

## Data Availability Statement

All datasets presented in this study are included in the article.

## Ethics Statement

Ethical review and approval was not required for the study on human participants in accordance with the Local Legislation and Institutional Requirements. The patients/participants provided their written informed consent to participate in this study.

## Author Contributions

QW performed most of the experiments and was a major contributor in writing the manuscript. DO, YK, MK, MAy, MAk, YA, HN, and KA interpreted the data and revised the manuscript. KY performed the experiments and acquired the data. TH and HM contributed to the study conception and design and revised the manuscript. All authors read and approved the final manuscript.

## Conflict of Interest

The authors declare that the research was conducted in the absence of any commercial or financial relationships that could be construed as a potential conflict of interest.
